# Bacteria make surgical strikes on host ubiquitin signaling

**DOI:** 10.1371/journal.ppat.1009341

**Published:** 2021-03-18

**Authors:** Tyler G. Franklin, Jonathan N. Pruneda

**Affiliations:** Department of Molecular Microbiology and Immunology, Oregon Health & Science University, Portland, Oregon, United States of America; Tufts Univ School of Medicine, UNITED STATES

## What are their motives?

Ubiquitin signaling is an essential eukaryotic posttranslational modification that regulates a gamut of cellular processes ranging from a classical role in proteasomal degradation to emerging roles in autophagy and immunity. Its breadth of signaling roles stems from the unique ability of ubiquitin to be assembled into complex poly-ubiquitin chains through any of 7 lysine residues or the amino terminus. Identifying the regulators and signaling outcomes of each type of poly-ubiquitin chain is an active area of research, but the emerging theme thus far is that distinct cellular messages are encoded in each linkage form [1].

Despite not encoding a functional ubiquitin system of their own, some pathogenic bacteria have evolved the remarkable ability to regulate discrete host poly-ubiquitin signals through the action of secreted effector proteins, providing them with a significant strategic advantage during infection. For example, the ability to induce the ubiquitin-dependent degradation of host response factors is an important component of *Shigella flexneri* infection [2–4]. Meanwhile, the ability of *Salmonella* Typhimurium to remove ubiquitin signals offers it a competitive advantage [5]. The evolutionary pressure to target host ubiquitin signaling is so strong that entirely convergent mechanisms of regulation have arisen, and in some cases, these methods of ubiquitin manipulation make up a sizeable proportion of a bacterium’s virulence factor repertoire [6].

### The bacterial arsenal

Ubiquitin is a 76–amino acid protein that is typically attached to lysine residues of target proteins after passing through an E1, E2, and E3 enzyme cascade. Classically, poly-ubiquitin chain specificity is determined by the last enzyme to form a labile cysteine linkage with the ubiquitin carboxyl terminus. In the case of RING-type E3 ligases, this means that chain specificity is encoded by the E2 ubiquitin-conjugating enzyme. HECT- or RBR-type E3 ligases, however, form one final cysteine linkage with ubiquitin and thus dictate chain specificity themselves. Bacterial pathogens such as *S*. *flexneri*, *S*. Typhimurium, *Legionella pneumophila*, and enterohemorrhagic *Escherichia coli* (EHEC) have all acquired E3 ligases that transfer ubiquitin through a cysteine-based mechanism and can dictate poly-ubiquitin chain specificity [7–10]. Remarkably, aside from some coarse similarities, these bacterial E3 ligases are structurally and mechanistically distinct from any analogous eukaryotic enzymes, suggesting convergent evolution of mechanisms for chain-specific poly-ubiquitin signaling.

In eukaryotes, ubiquitination is reversed through the action of proteases termed deubiquitinases that hydrolyze the (iso)peptide linkages of ubiquitin signals. Some deubiquitinases demonstrate exquisite poly-ubiquitin chain specificity, while others show more relaxed preferences or no chain specificity at all [11]. Bacterial deubiquitinases have been identified in a range of pathogens including *Salmonella*, *Legionella*, and *Chlamydia trachomatis* [5,12,13]. While some bacterial deubiquitinases distantly resemble examples in eukaryotes, others appear to have arisen through convergent evolution in order to manipulate discrete ubiquitin signals during infection [14–16].

In addition to modifying poly-ubiquitin signals directly, bacteria have also acquired methods to modulate the activities of host ubiquitin regulators and responders. In this way, bacteria can block specific ubiquitin signaling pathways or mask the signal from being read [17,18]. In fact, some of the posttranslational modifications that bacteria use to inactivate components of the ubiquitin system are entirely foreign to eukaryotic biology, essentially making them irreversible [18].

### Bacterial ligases destroy key targets

Poly-ubiquitin signals linked through Lys48 are the canonical message for proteasomal degradation, and bacterial E3 ligases frequently take advantage of this process to selectively degrade target host proteins [1]. Specificity for assembling the Lys48 poly-ubiquitin signal has been evolved by a range of structurally distinct folds, including the HECT-like (e.g., *Salmonella* SopA) and NEL (e.g., *Shigella* IpaH9.8) families of effector ligases, both of which depend upon a cysteine mechanism to facilitate direct ubiquitination of a target [7,8].

SopA from *Salmonella* uses a familiar HECT-like mechanism to assemble Lys48-linked poly-ubiquitin chains onto the host E3 ligases TRIM56 and TRIM65, which may be related to SopA’s role in enteritis [19,20]. The NEL family also assembles Lys48-linked poly-ubiquitin chains onto substrates, although NELs are more structurally and mechanistically distinct from any eukaryotic E3 ligases [21,22]. NELs are widely used by *Salmonella* and *Shigella*, which encode 3 and 12 family members, respectively. Aside from one report of Lys27 specificity [23], the majority of NELs are believed to be Lys48 specific and induce degradation of their targets. For example, *Salmonella* SspH1 has been shown to target the host serine/threonine kinase PKN1 in order to dampen the host inflammatory response to infection [7,24]. *Shigella* has evolved a remarkable expansion of NEL effectors, which provide a means to selectively target a number of host factors for degradation, including components of inflammatory signaling and cytosolic defense [3,4,23,25,26]. Notably, some bacterial ligases have been reported to target more than 1 host factor for ubiquitination, thus expanding their reach for host manipulation even further.

**Fig 1:**
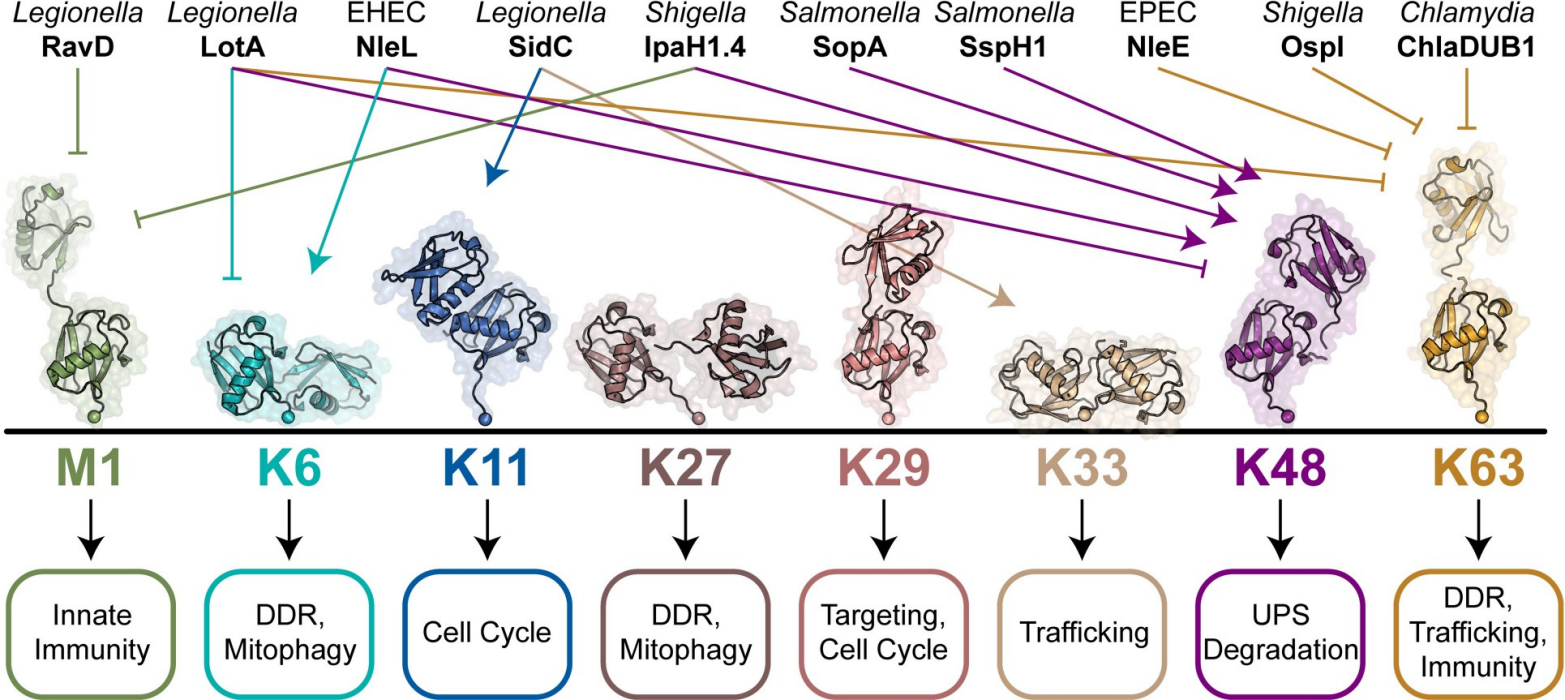
Bacteria manipulate specific poly-ubiquitin signals. Secreted bacterial effectors are shown positively or negatively regulating specific poly-ubiquitin signal types. Individual poly-ubiquitin chains are represented by their di-ubiquitin crystal structures (PDB codes 2W9N, 2XK5, 2XEW, 6QML, 4S22, 4XYZ, 5GOI, and 2JF5). Current models for the signaling roles of each poly-ubiquitin chain type are shown below. DDR, DNA damage response; UPS, ubiquitin–proteasome system.

Lys48-specific E3 ligases are a powerful and popular strategy of manipulating host responses, as they allow bacteria to tap into the ubiquitin–proteasome system for targeted protein degradation (Fig 1 and Table 1).

**Table 1:** Linkage-specific ubiquitin-targeted bacterial effectors.

Pathogen	Effector	Activity	Specificity	Target	Outcome	Reference
*Salmonella* Typhimurium	SopA	E3 Ligase	Lys48	TRIM56, TRIM65	Degradation	[8,19]
*S*. Typhimurium	SspH1	E3 Ligase	Lys48	PKN1	Degradation	[7,24]
*Shigella flexneri*	IpaH9.8	E3 Ligase	Lys48	GBPs, NEMO	Degradation	[3,4,23]
*S*. *flexneri*	IpaH4.5	E3 Ligase	Lys48	TBK1	Degradation	[25]
*S*. *flexneri*	IpaH0722	E3 Ligase	Lys48	TRAF2	Degradation	[26]
*S*. *flexneri*	IpaH1.4, 2.5	E3 Ligase	Lys48	HOIP	Degradation	[32]
*Legionella pneumophila*	SidC, SdcA	E3 Ligase	Lys11, 33	Vacuolar proteins	Remodeling	[9]
EHEC	NleL	E3 Ligase	Lys6, 48	JNK	Unknown	[10,33]
*L*. *pneumophila*	MavC	Ligase/Transglutaminase	Lys63	UBE2N	Signal inhibition	[27]
*L*. *pneumophila*	MvcA	DUB/Transglutaminase	Lys63	UBE2N	Signal activation	[28,29]
*S*. Typhimurium	SseL	DUB	Lys63	Various	Signal elimination	[5,14,31]
*Chlamydia trachomatis*	ChlaDUB1	DUB	Lys63	Various	Signal elimination	[13,14,30]
*L*. *pneumophila*	SdeA	DUB	Lys63	Vacuolar proteins	Signal elimination	[12]
*L*. *pneumophila*	RavD	DUB	Met1	Vacuolar proteins	Signal elimination	[16]
*L*. *pneumophila*	LotA	DUB	Lys6, 48, 63	Vacuolar proteins	Unknown	[34]
*S*. *flexneri*	OspI	Deamidase	Lys63	UBE2N	Signal inhibition	[17]
EPEC	NleE	Methyltransferase	Lys63	TAB2, TAB3	Signal masking	[18]

Compilation of the activities used by various bacterial effector proteins to either directly or indirectly manipulate specific poly-ubiquitin signal types and the direct outcomes of these interactions.

EHEC, enterohemorrhagic *Escherichia coli*; EPEC, enteropathogenic *Escherichia coli*.

### Bacteria cut off host communications

Beyond its role in targeted protein degradation, select poly-ubiquitin chain types can serve diverse signaling functions in, for example, immune signaling pathways. Innate immune signaling relies heavily upon several types of poly-ubiquitin signals. Cytokine and pattern recognition receptors often require the addition of Lys63-linked poly-ubiquitin chains to the receptor signaling complex for a downstream transcriptional response [1]. The generation of the Lys63-linked signal in these contexts requires the chain-specific E2 ubiquitin-conjugating enzyme UBE2N. To surgically block Lys63-linked poly-ubiquitin signaling pathways, *Shigella* has evolved the effector protein OspI that deamidates a key surface residue on UBE2N, leading to its inactivation and subsequently an impaired inflammatory response [17]. The activity of UBE2N is also tightly regulated by *Legionella* through the competing actions of MavC and MvcA, which catalyze the noncanonical (de)ubiquitination of UBE2N through a transglutamination reaction [27–29]. Downstream of Lys63-linked ubiquitination, TAB2 and TAB3 specifically recognize the Lys63-linked signal through ubiquitin-binding domains and activate TAK1. To block this step of inflammatory signaling, enteropathogenic *E*. *coli* (EPEC) has acquired NleE, a cysteine methyltransferase that modifies the ubiquitin-binding domains of TAB2 and TAB3, thereby blocking their ability to recognize Lys63-linked signals [18]. Another common strategy for interrupting Lys63 poly-ubiquitin signaling is through its specific reversal by bacterial deubiquitinases. The CE clan of bacterial deubiquitinases appear to have convergently evolved a preference for the hydrolysis of Lys63-linked chains, and these effectors have demonstrated roles in inhibiting inflammatory signaling, blocking autophagy, and maintaining the bacteria-containing vacuolar compartment in *Chlamydia*, *Salmonella*, and *Legionella*, respectively [12,14,30,31].

Met1-linked poly-ubiquitin chains also play roles in the innate immune response, often immediately downstream of Lys63-linked poly-ubiquitin signaling. These ubiquitin chains are solely assembled through the linear ubiquitin chain assembly complex (LUBAC) and play an important role in response to bacterial invasion [1]. As for Lys63-linked chains, *Shigella* has also developed a means to block the formation of Met1 poly-ubiquitin signals. The NELs IpaH1.4 and IpaH2.5 attach Lys48-linked poly-ubiquitin to the catalytic subunit of LUBAC, targeting it for proteasomal degradation and thereby preventing Met1 poly-ubiquitin chain formation and subsequent inflammatory signaling [32]. Using IpaH9.8, a separate NEL effector, *Shigella* also targets the Met1 poly-ubiquitin sensor protein NEMO for ubiquitin-dependent proteasomal degradation, thus blocking activation of the IκBα kinase complex required for NF-κB signaling [23]. Met1-linked poly-ubiquitin chains can also stimulate inflammatory signaling from the surface of a pathogen-containing vacuole. *Legionella* counteracts this by directing the Met1-specific deubiquitinase RavD to the cytosolic face of the *Legionella*-containing vacuole [16].

Thus, for both Lys63- and Met1-mediated signaling processes, bacteria have evolved unique strategies to specifically block a signal’s formation, mask its sensing, or remove it altogether (Fig 1 and Table 1).

### Bacterial code talkers transmit cryptic messages

For some poly-ubiquitin chain linkages such as Lys33 and Lys6, the specific regulators, substrates, and signaling outcomes are not fully understood [1]. Curiously, although many aspects of these so-called “atypical” poly-ubiquitin chains remain a mystery, bacteria appear to have selected for mechanisms that specifically interact with these signal types. *Legionella* has acquired a novel E3 ligase fold that uses a cysteine-dependent mechanism to assemble Lys11- and Lys33-linked poly-ubiquitin chains, which are proposed to remodel the *Legionella*-containing vacuole [9]. Although Lys11-linked signals are thought to be primarily degradative, the proposed functions of SidC and the related SdcA may be more congruent with the connection between Lys33-linked signals and protein trafficking. Lys6-linked poly-ubiquitin signals, which have been loosely tied to the DNA damage response and mitophagy, are also targeted during bacterial infection. EHEC, for example, encodes a HECT-like E3 ligase called NleL that assembles Lys48- and Lys6-linked chains, although the relevance of these specificities has not been tied to its role in regulating pedestal formation [10,33]. On the other hand, *Legionella* has acquired an effector protein called LotA that encodes dual deubiquitinase domains, one of which specifically hydrolyzes Lys6-linked signals at the surface of the *Legionella*-containing vacuole [34]. Why EHEC and *Legionella* have evolved opposing mechanisms to regulate Lys6-linked poly-ubiquitin and how these processes align with current models of this signal’s function remain unknown.

Given how little we understand about the roles and regulation of atypical poly-ubiquitin chains, it is interesting to consider what evolutionary pressures led to the acquisition of atypical linkage-specific effector proteins and how future research can leverage these enzymes to study human biology (Fig 1 and Table 1).

### Gathering strategic intelligence

From an evolutionary perspective, it is remarkable that bacteria have evolved unique strategies for manipulating the eukaryote-specific posttranslational modifier ubiquitin and even more astounding that they have gone to the lengths of targeting specific types of poly-ubiquitin signals so as to enact surgical strikes on cellular processes in the infected host. With mechanisms that are both familiar and foreign to our understanding of eukaryotic ubiquitin regulation, bacterial pathogens have the capability to tap into our system of targeted protein degradation, block our ability to signal and respond to infection, and manipulate certain poly-ubiquitin signals that we don’t yet fully understand. Additional work at this complex host–pathogen interface has the potential to not only provide strategic insight into bacterial pathogenesis and mechanisms of disease, but also explain cryptic facets of human ubiquitin signaling.
